# Erdafitinib treatment in metastatic urothelial carcinoma: a real-world analysis

**DOI:** 10.3389/fonc.2023.1151701

**Published:** 2023-05-24

**Authors:** Keren Rouvinov, Eran Levanon, Avivit Peer, Michal Sarfaty, David Sarid, Victoria Neiman, Eduard Grikshtas, Eli Rosenbaum, Igal Kushnir, Barak Talmor, Michael Friger, Yonaton Zarbiv, Eli Gez, Hadas Dresler, Walid Shalata, Amichay Meirovitz, Noa Shani Shrem, Alexander Yakobson, Wilmosh Mermershtain, Daniel Keizman

**Affiliations:** ^1^The Legacy Heritage Oncology Center & Dr. Larry Norton Institute, Soroka Medical Center, Beer Sheva, Israel; ^2^Ben-Gurion University of the Negev, Beer Sheva, Israel; ^3^Faculty of Health Science, Ben-Gurion University of the Negev, Beer Sheva, Israel; ^4^Department of Oncology, Rambam Medical Center, Haifa, Israel; ^5^Department of Oncology, Sheba Medical Center, Tel Aviv, Israel and Sackler Faculty of Medicine, Tel Aviv University, Tel Aviv, Israel; ^6^Department of Oncology, Tel Aviv Sourasky Medical Center Affiliated to the Sackler School of Medicine, Tel-Aviv University, Tel-Aviv, Israel; ^7^Department of Oncology, Rabin Medical Center, Petah Tikva, Israel; ^8^Department of Oncology, Lin Medical Center, Haifa, Israel; ^9^Department of Oncology, Meir Medical Center, Kfar Saba, Israel and Sackler Faculty of Medicine, Tel Aviv University, Tel Aviv, Israel; ^10^Department of Oncology, Hadassah Medical Center, Jerusalem, Israel; ^11^Department of Oncology, Assuta Medical Center, Ashdod, Israel; ^12^Department of Oncology, Shaare Zedek Medical Center, Jerusalem, Israel

**Keywords:** erdafitinib, metastatic urothelial carcinoma, treatment, real-world analysis, fibroblast growth factor receptor (FGFR) inhibitor

## Abstract

**Background:**

Erdafitinib, a fibroblast growth factor receptor (FGFR) inhibitor is a standard post chemotherapy advanced treatment line for metastatic urothelial carcinoma harboring FGFR2/3 genomic alterations. It was approved based on a phase 2 clinical trial, revealing a 40% response rate, and 13.8 months overall survival. These FGFR genomic alterations are uncommon. Thus, real-world data on erdafitinb use is scant. We herein describe erdafitinib treatment outcome in a real world patient cohort.

**Methods:**

We retrospectively reviewed the data of patients treated with erdafitinib from 9 Israeli medical centers.

**Results:**

Twenty-five patients with metastatic urothelial carcinoma (median age 73, 64% male, 80% with visceral metastases) were treated with erdafitinib between January 2020 to October 2022. A clinical benefit (complete response 12%, partial response 32%, stable disease 12%) was seen in 56%. Median progression-free survival was 2.7 months, and median overall survival 6.73 months. Treatment related toxicity ≥ grade 3 occurred in 52%, and 32% discontinued therapy due to adverse events.

**Conclusions:**

Erdafitinib therapy is associated with a clinical benefit in the real world setting, and associated with similar toxicity as reported in prospective clinical trials.

## Introduction

Urothelial carcinoma is the second most common urological malignancy in the western world (1). Until recent years, the standard of care in the metastatic setting consisted only of platinum based chemotherapy in the first line setting, with limited activity (10% response rate and short overall survival) of chemotherapy as advanced treatment line (2–4). In 2017, immune checkpoint inhibitors were incorporated in the treatment paradigm, mainly in the post platinum based chemotherapy setting, but are associated with a durable clinical benefit only in a minority of patients (20% response rate) (1, 2, 5). Recently, two more therapies were FDA approved for these patients in the advanced treatment line, the FGFR inhibitor erdafitinib, and the antibody-drug conjugates enfortumab vedotin (demonstrating improvement of overall survival) and sacituzumab govitecan (based on a phase 2 trial) (6–8).

The FGFR signaling pathway plays a key role in several tumorigenesis cellular processes, such as proliferation, survival, migration, differentiation, and angiogenesis (9). Erdafitinib is an oral tyrosine kinase inhibitor of fibroblast growth factor receptors (FGFR), that inhibits FGF activity by binding to FGFR 1-4 (3, 10).

The open–label, phase II BLC2001 trial, assessed the efficacy of erdafitinib in 99 patients with metastatic urotherlial carcinoma (mUC) progressing after platinum based chemotherapy, and harboring a genomic alteration in FGFR 2–3, including FGFR3 mutation or FGFR2/3 fusion. It revealed a response rate (RR) of 40%, median progression free survival (PFS) of 5.5 months, and an overall survival (OS) of 13.8 months. Grade 3–4 adverse events occurred in 67% of patients, most commonly hyponatremia, stomatitis and asthenia (3).

Based on this study, erdafitinib was FDA approved as standard of care in mUC with FGFR 2/3 genomic alterations, that progressed following platinum–based chemotherapy, including within 12 months of neoadjuvant or adjuvant treatment (4).

Only a minority of patients (up to 20% overall, and more common in upper tract UC versus urinary bladder origin) with mUC harbor FGFR 2/3 genomic alterations (3). Thus, despite being an approved standard of care, there is limited real world data regarding erdafitinib therapy in this setting. In the present study we aimed to report real world outcome of erdafitinib therapy in mUC.

## Patients and methods

### Study group

This was a retrospective multicenter cohort study. It included patients with mUC, and FGFR 2/3 genomic alterations, as detected by next generation sequencing of tumor sample. Patients were treated with erdafitinib between January 2020 to October 2022 in 9 Israeli medical centers, including Assuta, Hadassah, Lin, Meir, Rabin, Rambam, Sheba, Soroka, Tel–Aviv Sourasky. Patient data were retrospectively collected from electronic medical records and paper charts and included the following clinicopathologic parameters: age, male versus female, number and type of previous treatment lines, histology subtypes, ECOG performance status, smoking status (active, past, never), treatment of the primary tumor by surgery or radiotherapy, metastatic sites, and pre–treatment laboratory values.

### Erdafitinib therapy

Before erdafitinib treatment initiation, all patients had clinical and radiologic disease progression. Erdafitinib was administered orally, usually with a starting dose of 8 mg once a day, and in selected patients with significant comorbidities or poor performance status, at a reduced dose. If possible, subsequent dose increase to 9 mg/day was done as per standard guidelines. Dose reduction or treatment interruption due to adverse events were done according to standard practice. Treatment was continued until disease progression, unacceptable adverse events or death. Patient follow–up consisted of regular physical examinations and laboratory assessments every 4–6 weeks and imaging studies every 12–16 weeks.

### Treatment outcomes

For the evaluation of response, the Response Evaluation Criteria in Solid Tumors (RECIST) version 1.1 was applied (11). The response was assessed by independent radiologists and treating physicians. Duration of treatment was defined as the time from erdafitinib treatment initiation until treatment discontinuation. Overall survival was defined as the time from the initiation of treatment to death of any cause.

### Statistical analysis

Patients who did not progress on treatment, or die by December 2022 were censored in the treatment duration and overall survival analyses. Univariate analysis (unadjusted) was used to analyze the association between outcomes and pre–erdafitinib treatment clinicopathologic factors, by logistic regression for response rate, and the Cox regression model for survival outcome. Survival probabilities and median survival times were estimated from Kaplan–Meier curves. Data were analyzed using SPSS software (SPSS for Windows, USA).

### Regulatory considerations

The research was approved by the institutional review ethics boards of the institutions involved in the study.

## Results

### Patient characteristics

Study group included twenty–five patients with mUC that were treated with erdafitinib. Median age was 73 years, and 64% were male. The primary tumor of origin was upper tract in 56% (n=14, 11 with renal pelvic tumor, and 3 with ureteral tumor), and urinary bladder in 44% (n=11). All patients had pure urothelial carcinoma (no patient with mixed or variant histology). Fourteen patients (56%) were initially diagnosed with a non metastatic primary tumor, treated with surgery (n=13) or radiation (n=1). Ten patients (44%) were diagnosed upfront with metastatic disease.

Twenty–one patients (80%) had visceral metastases. ECOG performance status was 0–1 in 64% (n=16), and 2 in 36% (n=9).

### FGFR2/3 genomic alterations

84% (n=21) had an FGFR3 mutation, and 12% (n=3) an FGFR2–3 fusion, in one patient the specific genomic alteration was unknown. Data regarding the specific FGFR alteration is included in **Table 1**.

**Table 1:** FGFR 2,3 genetic alterations.

Type of FGFR 2,3 genetic alterations	Value n(%)
FGFR3 mutation	21 (84%)
p.S249C	13 (52%)
p.R248C	1 (4%)
p.G370C	2 (8%)
p.Y373C	1 (4%)
unknown type of FGFR3 mutation	4 (16%)
FGRF2,3 fusion	3 (12%)
FGFR3-TACC3	2 (8%)
FGFR3-TACC3v1	1 (4%)
Unknown	1 (4%)

### Erdafitinib treatment initiation

Erdafitinib was given as first line therapy in 8% (n=2), second line in 44% (n=11), third line in 44% (n=11), and forth line in 4% (n=1). In terms of prior therapies, in 20% (n=5) it consisted of platinum based chemotherapy only, 32% (n=8) prior immunotherapy (pembrolizumab) only, and 40% (n=10) both prior platinum based chemotherapy and immunotherapy (pembrolizumab). Pre–erdafitinib chemotherapy consisted of the regimens gemcitabine and cisplatin, gemcitabine and carboplatin, and dose dense MVAC.

Patient pre–treatment characteristics are presented in **Table 2**.

**Table 2:** Erdafitinib Pre-treatment patient characteristics.

Factors	Distribution	Univariate analysis for DFS p-value
Age (yr), median (range)	73 (52-87)	0.7
Female vs Male		0.66
Female	36% (n=9)	
Male	64% (n=16)	
ECOG		0.491
0	16% (n=4)	
1	48% (n=12)	
2	36% (n=9)	
Number of prior lines		0.53
0	8% ( n=2)	
1	44% (n=11)	
2	44% (n=11)	
3	4% (n=1)	
Primary tumor location		0.045
Upper tract	56% (14)	
Lower tract	44% (11)	
Creatinine clearance rate		0.361
<60mL/min	32% (8)	
>60mL/min	48% (12)	
Hemoglobin level g/dl		0.165
>10		
<10		
Metastatic site		
Lung	64% (16)	0.012
Bone	16% (4)	
Liver	28% (7)	
Lymph node	52% (13)	
Brain	4% (1)	
FGFR alterations		
FGFR3 mutation	84% (21)	
FGRF2,3 fusion	12% (3)	
Unknown	4% (1)	

Initial dose of erdafitinib was standard 8mg in 60% (n=15), and reduced in 40% (n=10), d/t decreased performance status and significant comorbidities.

After treatment initiation, standard dose increase to 9mg was done in 24% (n=6).

### Erdafitinib treatment outcomes

**Figure 1:**
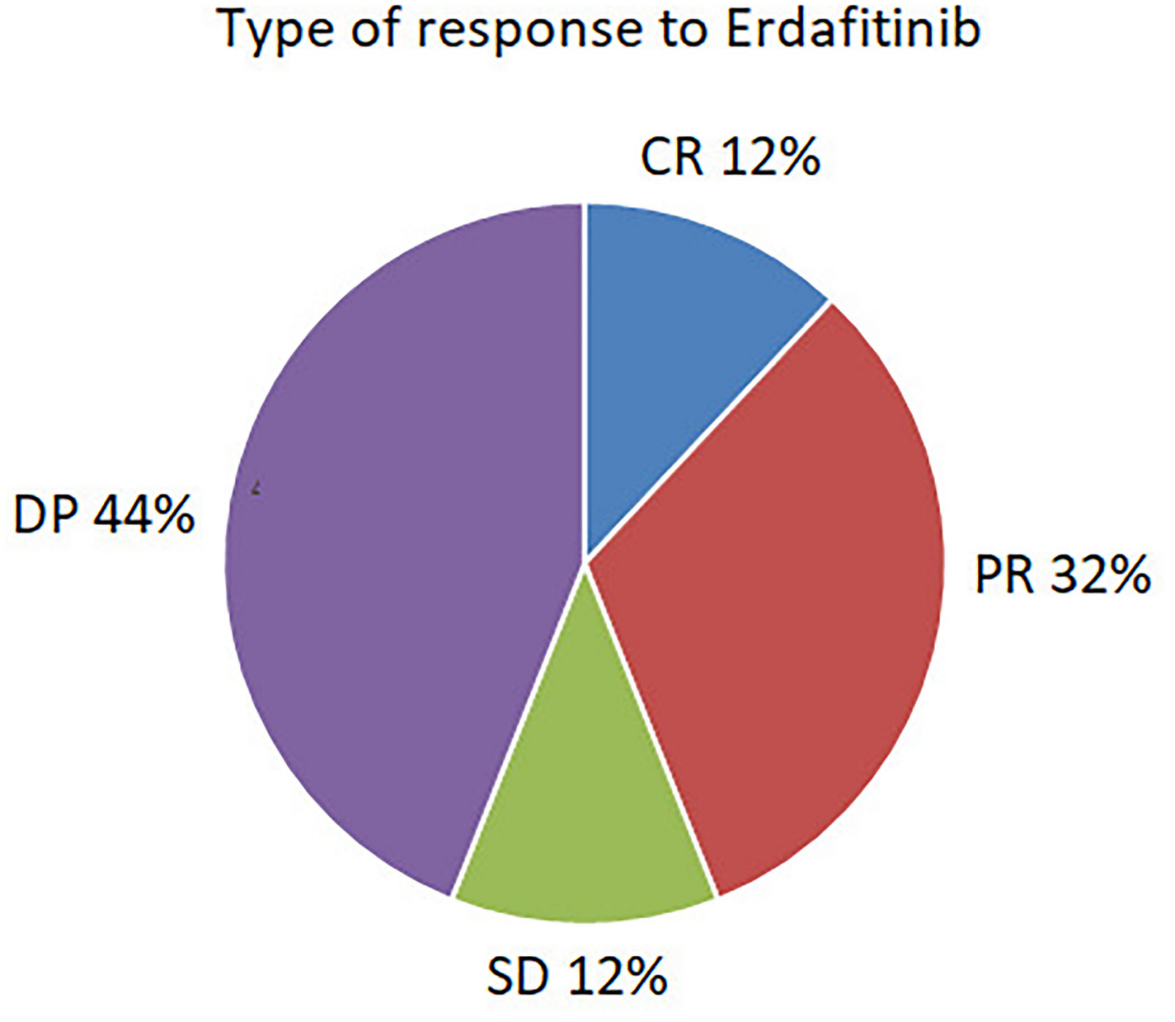
Type of response to Erdafirinib.

Median follow–up time was 24 months (range 13–30 months). A clinical benefit was seen in 56% (n=15), consisting of complete response in 12% (n=3), partial response in 32% (n=8), and stable disease in 12% (n=3). 44% (n=11) were refractory to treatment. The type of response is depicted in **Figure 1**.

Erdafitinib treatment outcomes are presented in **Table 3**.

**Table 3:** Erdafitinib treatment outcomes.

Type of response	Value n (%)
Best overall response	
Complete response	3 (12%)
Partial response	8 (32%)
Stable disease	3 (12%)
Progressive disease	11 (44%)

**Figure 2:**
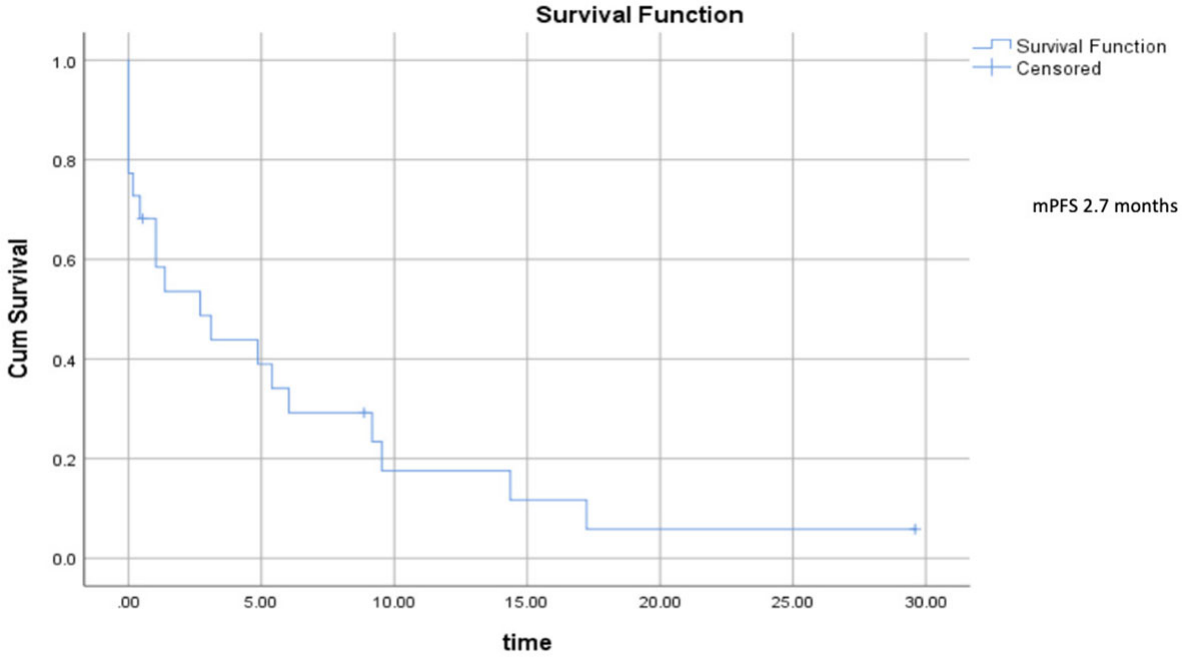
Progression–free survival.

**Figure 3:**
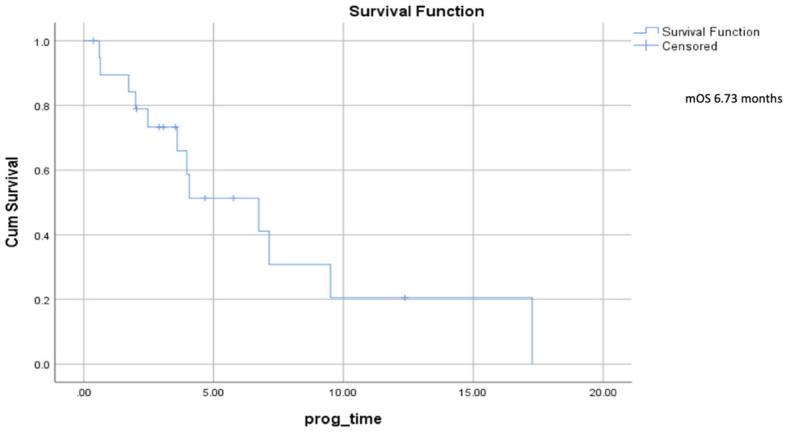
Overall survival.

Median progression–free survival was 2.7 months (range 0.5–5.7) (**Figure 2**), and median overall survival was 6.73 months (range 2.5–10.9) (**Figure 3**).

**Figure 4:**
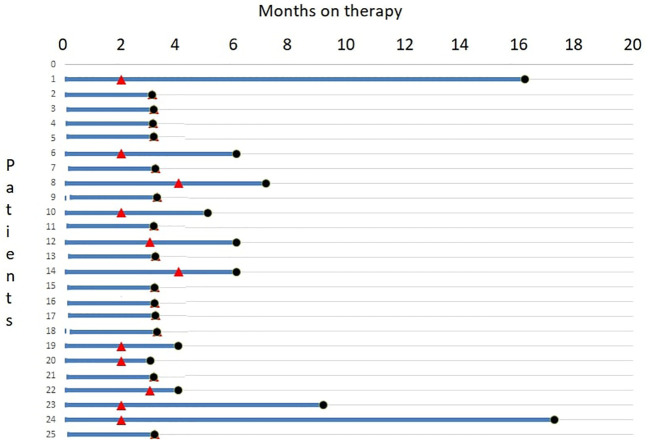
A swimmer plot of therapy response and duration.

A swimmer plot of therapy response and duration is depicted in **Figure 4**.

### Factors associated with progression–free survival

In univariate analysis, tumor location (upper tract versus bladder, p=0.045) and liver metastases (no versus yes, p=0.012) were associated with progression– free survival. None of the following factors were associated with progression–free survival: male versus female, p=0.627, ECOG status (0–1 versus 2, p=0.491), number of previous treatment lines (0–1 versus 2 or more, p=0.53), smoking status (p=0.473), pre–treatment levels of hemoglobin (p=0.165), pre–treatment estimated creatinine clearance (p=0.361).

In univariate analysis, no factor was associated with overall survival.

### Erdafitinib treatment associated toxicity

Treatment associated adverse events are presented in **Table 4**. The most common were hyperphosphatemia in 56% (n=14), mucositis in 32% (n=8), fatigue in 28% (n=7), diarrhea in 20% (n=5), and palmar–plantar erythrodysesthesia syndrome in 12% (n=3). The most common grade 3 or 4 toxicities were mucositis in 16% (n=4), fatigue in 16% (n=4), and diarrhea in 8% (n=2). 32% (n=8) permanently discontinued erdafitinib due to severe grade 3–4 toxicity.

**Table 4:** Erdafirinib treatment associated adverse events.

Adverse event	All grade, n (%)	Grade 3 or 4, n (%)
Mucositis	8 (32)	4 (16)
Fatigue	7 (28)	4 (16)
Hyperphosphatemia	14 (56)	1 (4)
Diarrhea	5 (20)	2 (8)
Palmar-plantar Erythrodysesthesia syndrome	3 (12)	0 (0)
Onycholysis	1 (4)	1 (4)
Nail disorder	2 (8)	1 (4)
Dry eye	1 (4)	0 (0)
Vision disturbance	2 (8)	0 (0)
Dry mouth	2 (8)	0 (0)
Acute renal failure	1 (4)	0 (0)
Hypercalcemia	1 (4)	0 (0)
Muscle pain	1 (4)	0 (0)
Dyspnea	1 (4)	0 (0)
Decreased appetite	1 (4)	0 (0)
Sepsis	1 (4)	0 (0)
Leg edema	1 (4)	0 (0)

## Discussion

In the present study of real world setting, erdafitinib therapy in patients with mUC was associated with a clinical benefit (in 56% of patients) and similar toxicity as reported in prospective clinical trials.

Based on the BLC2001 phase 2 clinical trial, erdafitinib was FDA approved as a standard advanced line of therapy in mUC with FGFR 2/3 genomic alterations, that progressed during or following previous platinum–based chemotherapy (4). However, only a minority of patients (up to 20% overall, more common in upper tract tumors) have FGFR 2/3 genomic alterations eligible for therapy (3). Thus, despite being an approved standard of care, there is limited real world data regarding its efficacy.

In the present study of real world setting, erdafitinib therapy was associated with a clinical benefit in 56% of patients, median progression–free survival of 2.7 months, median overall survival of 6.73 months, and known toxicity (similar previous clinical trials).

The efficacy of erdafitinib therapy seen in the present study is similar to a Brazilian real life prospective study of an expanded access program (1), where erdafitinib was given as third line of therapy, and associated with a clinical benefit (response or stable disease) of 50% and a median time to treatment failure was 2.8 months.

The progression free survival and overall survival in the present study were shorter than those reported in the BCL2001 prospective trial (3, 4). This may be due to the fact that a significant proportion of patients in the present study were treated with erdafitinib in a more advanced line setting (48% treated as third or fourth–line of therapy), similar to the Brazilian real life prospective study (1), and 40% of them were treated after both chemotherapy and immunotherapy. Furthermore, 36% had a decreased ECOG performance status of 2, and most were with high volume disease (80% with visceral metastases, and 4% with brain metastases).

In the present study, significantly more patients discontinued treatment due to adverse events compared to those in pivotal BCL2001 trial (32% versus 13%). This might correlate with the generally more unfit population in the real–world setting.

The present study has limitations. First, its retrospective nature may be associated with known biases. Second, the study cohort is small (as other real world reports due to the fact that eligible FGFR genomic alterations are uncommon). Third, the present study patient population is unselected and heterogeneous (e.g various number of previous treatment lines). We therefore can’t exclude that unequal distribution of unidentified clinicopathologic parameters may have biased the observed results. Furthermore, at present, we do not have data on resistance mechanisms or immune status that could explain the poorer outcome of patients in the present study, or the diffence in outcome between upper tract vs bladder tumors (in the present study the proportion of upper tract tumors was higher than in the pivotal BLC2001 trial, 56% vs 20%).

Nonetheless, we feel that this real world data report is important, since it reveals the clinical benefit of erdafitinib in heavily pretreated patients, including those with a reduced performance status, comorbidities and brain metastases, which usually are not included in prospective clinical trials. Future larger studies are needed to confirm our results.

In conclusion, despite the approval of erdafitinib as advanced treatment line for metastatic urothelial carcinoma, real–world data on this therapy is limited. The present study suggests that erdafitinib therapy is associated with clinical benefit in there real world setting, including in heavily pre–treated patients.

## Data availability statement

The original contributions presented in the study are included in the article/supplementary material. Further inquiries can be directed to the corresponding author.

## Ethics statement

The studies involving human participants were reviewed and approved by Helsinki clalit committee. Written informed consent for participation was not required for this study in accordance with the national legislation and the institutional requirements.

## Author contributions

KR, EL, and DK contributed to conception and design of the study. KR, EL, MS, DS, VN, EG, ER, IK, BT, YZ, EIG, HD, WS, AM, NSS, AY and WM organized the database. MF performed the statistical analysis. EL and KR wrote the first draft of the manuscript. KR, EL, AP, and DK wrote sections of the manuscript. All authors contributed to the article and approved the submitted version.
